# The Role of *Enterococcus faecium* as a Key Producer and Fermentation Condition as an Influencing Factor in Tyramine Accumulation in *Cheonggukjang*

**DOI:** 10.3390/foods9070915

**Published:** 2020-07-11

**Authors:** Young Kyoung Park, Young Hun Jin, Jun-Hee Lee, Bo Young Byun, Junsu Lee, KwangCheol Casey Jeong, Jae-Hyung Mah

**Affiliations:** 1 Department of Food and Biotechnology, Korea University, 2511 Sejong-ro, Sejong 30019, Korea; eskimo@korea.ac.kr (Y.K.P.); younghoonjin3090@korea.ac.kr (Y.H.J.); bory92@korea.ac.kr (J.-H.L.); by-love23@hanmail.net (B.Y.B.); jpang@korea.ac.kr (J.L.); 2 Department of Animal Sciences, University of Florida, Gainesville, FL 32611, USA; kcjeong@ufl.edu; 3 Emerging Pathogens Institute, University of Florida, Gainesville, FL 32611, USA

**Keywords:** *Cheonggukjang*, *Enterococcus faecium*, tyramine, biogenic amines, fermentation temperature, fermentation duration, tyrosine decarboxylase gene (*tdc*)

## Abstract

The study evaluated the role of *Enterococcus faecium* in tyramine production and its response to fermentation temperature in a traditional Korean fermented soybean paste, *Cheonggukjang*. Tyramine content was detected in retail *Cheonggukjang* products at high concentrations exceeding the recommended limit up to a factor of 14. All retail *Cheonggukjang* products contained *Enterococcus* spp. at concentrations of at least 6 Log CFU/g. Upon isolation of *Enterococcus* strains, approximately 93% (157 strains) produced tyramine at over 100 µg/mL. The strains that produced the highest concentrations of tyramine (301.14–315.29 μg/mL) were identified as *E*. *faecium* through 16S rRNA sequencing. The results indicate that *E. faecium* is one of the major contributing factors to high tyramine content in *Cheonggukjang*. During fermentation, tyramine content in *Cheonggukjang* groups co-inoculated with *E. faecium* strains was highest at 45 °C, followed by 37 °C and 25 °C. The tyramine content of most *Cheonggukjang* groups continually increased as fermentation progressed, except groups fermented at 25 °C. At 45 °C, the tyramine content occasionally exceeded the recommended limit within 3 days of fermentation. The results suggest that lowering fermentation temperature and shortening duration may reduce the tyramine content of *Cheonggukjang*, thereby reducing the safety risks that may arise when consuming food with high tyramine concentrations.

## 1. Introduction

*Cheonggukjang* is a traditional Korean soybean paste produced by fermenting soybeans with *Bacillus subtilis*. Traditional methods of *Cheonggukjang* production utilize rice straw added to steamed soybeans for a short fermentation period of approximately 2–3 days, while starter cultures are used instead of rice straw for modern methods of production [1,2]. Fermentation of *Cheonggukjang* is a process involving microbial enzymatic proteolysis resulting in uniquely characteristic savory aromatic and flavor properties [3]. Consumption of *Cheonggukjang* has been reported to be associated with numerous benefits such as antioxidative, antihypertensive, thrombolytic, and antimicrobial properties [4,5]. However, despite the beneficial properties of *Cheonggukjang*, potentially hazardous biogenic amines (BAs) may be produced during fermentation of the proteinous food rich in precursor amino acids.

The majority of BAs are formed through the reductive amination of ketones and aldehydes, as well as the decarboxylation of amino acids by microbially produced enzymes [6]. Though BAs are essential for the regulation of protein synthesis, nucleic acid functions, and membrane stabilization in living cells, consumption of food products with high concentrations of BAs may result in toxicological effects [7,8,9,10]. The excessive intake of food products such as mackerel, pacific saury, sardines, and tuna may result in “scombroid poisoning” owing to potentially high concentrations of toxic histamine that may cause symptoms similar to an allergic reaction including diarrhea, dyspnea, headache, hives, and hypotension [10,11,12,13]. Overconsumption of foods with high concentrations of tyramine may potentially result in a “cheese crisis” with various symptoms including heart failure, hemorrhages, hypertensive crisis, high blood pressure, and severe headaches [9,10,14,15]. Such a high content of tyramine produced by microbial tyrosine decarboxylase activity has occasionally been found in tyrosine-rich foods such as cheese [16,17] and soybean-based fermented products [18,19,20]. Therefore, Ten Brink, et al. [21] suggested BA toxicity limits of 30 mg/kg for *β*-phenylethylamine, 100 mg/kg for histamine, and 100–800 mg/kg for tyramine in foods.

Previous studies by Ko, et al. [18], Jeon, et al. [19], and Seo, et al. [20] on the BA content of *Cheonggukjang* have shown that tyramine in particular has been detected in high concentrations up to 1913.51, 251.66, and 905.0 mg/kg, respectively. Ibe, et al. [22] suggested that *Enterococcus faecium* may be largely responsible for the BA content of *Miso* (a Japanese fermented soybean paste). Notably, numerous studies have reported that *Enterococcus* spp. possess the tyrosine decarboxylase gene (*tdc*) [23,24]. Moreover, in particular Kang and Park [25] and Kang, et al. [26] confirmed the presence of *E. faecium* in *Cheonggukjang*, while a previous study by Jeon, et al. [19] showed that *Enterococcus* spp. isolated from *Cheonggukjang* exhibited tyramine production at concentrations of at least 351.59 μg/mL. Taken together, the previous reports imply that *E. faecium* may also be responsible for the BA content of *Cheonggukjang*. Meanwhile, the growth of *Enterococcus* spp. has been reported to occur at temperatures ranging from 10 °C up to 45 °C that overlap with *Cheonggukjang* fermentation temperatures ranging from 25 to 50 °C [27,28,29,30]. The corresponding range in temperature may be beneficial for *E. faecium* growth and tyramine production during the fermentation of contaminated *Cheonggukjang* products. Furthermore, a previous study reported that tyramine content increases in fermented soybeans as fermentation progresses [19]. According to Bhardwaj, et al. [31], the production of tyramine by *E. faecium* strains may be affected by incubation conditions such as temperature and time. Therefore, the current study assessed the safety risk of BAs (particularly tyramine) in *Cheonggukjang*, clarified the microorganism responsible for tyramine accumulation, and evaluated the effect of fermentation temperature/duration on *E. faecium* growth and subsequent tyramine production in the food.

## 2. Materials and Methods

### 2.1. Cheonggukjang Products

Six representative, but different *Cheonggukjang* products were purchased from various retail markets in the Republic of Korea and stored at 4 °C until further experimentation. Within a day of storage, the BA content of *Cheonggukjang* products was measured, followed by physicochemical and microbial analyses.

### 2.2. Physicochemical Analyses

To investigate the influencing factors such as pH, salinity, and water activity on BA content in *Cheonggukjang*, the physicochemical properties of *Cheonggukjang* samples (retail *Cheonggukjang* products purchased and *Cheonggukjang* groups fermented in this study) were measured as described below. Samples weighing 10 g using an analytical balance (Ohaus Adventurer™, Ohaus Corporation, Parsippany, NJ, USA) were homogenized with 90 mL of distilled water using a stomacher (Laboratory Blender Stomacher 400, Seward, Ltd., Worthing, UK). The pH of the homogenates was measured using a pH meter (Orion 3-star pH Benchtop Thermo Scientific, Waltham, MA, USA), while salinity was measured using the procedure described by the Association of Official Analytical Chemists (AOAC; Official Method 960.29) [32]. The water activity of the samples was measured using an electric hygrometer (AquaLab Pre, Meter Group, Inc., Pullman, WA, USA).

### 2.3. Microbial Analyses

The analysis of the microbial community in *Cheonggukjang* samples was conducted using Plate Count Agar (PCA; Difco, Becton Dickinson, Sparks, MD, USA); de Man, Rogosa, and Sharpe (MRS; Conda, Madrid, Spain) agar; and m-Enterococcus Agar (m-EA; MB Cell, Seoul, Korea) for the enumeration of total mesophilic viable bacteria, lactic acid bacteria, and *Enterococcus* spp., respectively. Samples weighing 10 g were homogenized with 90 mL of sterile 0.1% peptone saline using a stomacher. The homogenates were 10-fold serially diluted with 0.1% peptone saline up to 10^−5^, and 100 μL of each dilution was spread on PCA, MRS agar, and m-EA in duplicates. Incubation conditions were set according to the manufacturer’s instructions: PCA at 37 °C for 24 h and m-EA at 37 °C for 48 h under aerobic condition; MRS agar at 37 °C for 48 h under anaerobic condition. Anaerobic condition was achieved using an anaerobic chamber (Coy Lab. Products, Inc., Grass Lake, MI, USA) containing an atmosphere of 95% nitrogen and 5% hydrogen. After incubation, the bacterial concentrations of the *Cheonggukjang* samples were calculated by enumerating the colony-forming units (CFU) on the plates of respective media with approximately 10 to 300 colonies [33] and adjusting for the dilution.

### 2.4. Isolation and Identification of Enterococcus Strains from Retail Cheonggukjang Products

A total of 169 *Enterococcus* strains were isolated from retail *Cheonggukjang* products according to the method described by Mareková, et al. [34], with minor modifications. Upon enumeration of colonies on m-EA, individual colonies were streaked on MRS agar and incubated at 37 °C for 48 h under anaerobic condition. Single colonies were streaked again on MRS agar and incubated under the same conditions. The pure single colonies were inoculated in MRS broth, incubated at 37 °C for 48 h, and stored at −70 °C using glycerol (20%, *v*/*v*).

The identities (at species level) of the individual *Enterococcus* strains that displayed the highest tyramine production were further investigated through sequence analysis of 16S rRNA gene amplified with the universal bacterial primer pair 518F (5′-CCAGCAGCCGCGGTAATACG-3′) and 805R (5′-GACTACCAGGGTATCTAAT-3′) (Solgent Co., Daejeon, Korea). The identities of sequences were determined using the basic local alignment search tool (BLAST) of the National Center for Biotechnology Information (NCBI; http://www.ncbi.nlm.nih.gov/BLAST/).

### 2.5. Preparation of Cheonggukjang

To investigate the effect of fermentation temperature on tyramine production by *E. faecium*, several temperatures were set for in situ *Cheonggukjang* fermentation experiments. The temperature for *Cheonggukjang* fermentation (intermediate-temperature group) was determined based upon previous studies in which 37 °C was reported as the temperature commonly used for *Cheonggukjang* production [19,35,36]. In addition, the temperatures of 25 °C and 45 °C used by other studies for *Cheonggukjang* fermentation were utilized for the low and high temperature groups, respectively [29,30].

White soybeans (*Glycine* max Merrill) were purchased from a retail market in the Republic of Korea. The soybeans were soaked in distilled water at 4 °C for 12 h, and subsequently drained for 1 h. Approximately 200 g of soybeans were adjusted to a final salinity of 2.40% according to the salinity of *Cheonggukjang* outlined in the 9th revision of the Korean food composition table [37] and subsequently steamed at 125 °C for 30 min using an autoclave. The steamed soybeans were cooled to 50 °C and inoculated with bacterial inocula in M/15 Sörensen’s phosphate buffer (pH 7) to final concentrations of approximately 6 Log CFU/g of *B. subtilis* KCTC 3135 (also designated as ATCC 6051; type strain) and 4 Log CFU/g of *E. faecium* KCCM 12118 (ATCC 19434; type strain) or *E. faecium* CJE 216 (strain isolated from *Cheonggukjang* and selected owing to both strong tyramine production and *tdc* gene expression). The control group (without any *E. faecium* strains) was inoculated with only *B. subtilis* KCTC 3135 to a final concentration of 6 Log CFU/g. The sizes of inocula were selected with consideration of the cell count of each microorganism in *Cheonggukjang* products determined in our previous study [19]. The inoculated steamed soybeans were then fermented at 25 °C, 37 °C, or 45 °C for 3 days. Approximately 20 g of the fermented soybeans were collected daily during fermentation to measure the BA content as well as physicochemical and microbial properties. Fermented soybeans sampled during fermentation were stored at −70 °C for further testing, as required.

### 2.6. BA Analyses in Cheonggukjang Samples and Bacterial Cultures

#### 2.6.1. BA Extraction from *Cheonggukjang* Samples and Bacterial Cultures

Quantification of the BA content of *Cheonggukjang* was conducted as previously described by Ben-Gigirey, et al. [38]. Five grams of *Cheonggukjang* with 20 mL of 0.4 M perchloric acid (Sigma-Aldrich, St. Louis, MO, USA) were homogenized by vortex (Vortex-Genie, Scientific industries, Bohemia, NY, USA) and stored at 4 °C for 2 h. The mixture was then centrifuged at 3000× *g* for 10 min at 4 °C (1736R, Labogene, Seoul, Korea), and the supernatant was collected. Upon resuspension of the pellet with 20 mL of 0.4 M perchloric acid, the mixture was stored at 4 °C for 2 h and centrifuged again at 3000× *g* at 4 °C for 10 min. The supernatant was combined with the previously collected supernatant and adjusted to a final volume of 50 mL with 0.4 M perchloric acid. Then, the extract was filtered through Whatman paper No. 1 (Whatman International Ltd., Maidstone, UK).

The bacterial production of BAs was measured using the procedures described by Eerola, et al. [39], modified by Ben-Gigirey, et al. [38,40], and further modified in the present study to culture *Enterococcus* spp. based on Marcobal, et al. [41]. A loopful (10 µL) of glycerol stock of each enterococcal strain was inoculated in 5 mL of MRS broth supplemented with 0.5% of each amino acid, including L-histidine monohydrochloride monohydrate, L-tyrosine disodium salt hydrate, L-ornithine monohydrochloride, L-lysine monohydrochloride (pH 5.8), and 0.0005% pyridoxal-HCl (all from Sigma-Aldrich) and incubated at 37 °C for 48 h. Approximately 100 μL of the broth culture was then transferred to another tube containing 5 mL of the same medium. Upon incubation at 37 °C for 48 h, the broth culture was filtered using a sterile syringe with a 0.2 μm membrane (Millipore Co., Bedford, MA, USA). Then, 9 mL of 0.4 M perchloric acid were added to 1 mL of the filtered broth culture and mixed by a vortex mixer. The mixture was reacted in a cold chamber at 4 °C for 2 h and centrifuged at 3000× *g* at 4 °C for 10 min. The extract was filtered through Whatman paper No. 1.

#### 2.6.2. Preparation of Standard Solutions for High Performance Liquid Chromatography (HPLC) Analysis

Standard solutions with concentrations of 0, 10, 50, 100, and 1000 ppm were prepared for tryptamine, *β*-phenylethylamine hydrochloride, putrescine dihydrochloride, cadaverine dihydrochloride, histamine dihydrochloride, tyramine hydrochloride, spermidine trihydrochloride, and spermine tetrahydrochloride (all from Sigma-Aldrich). Internal standard solution with the same concentrations was prepared using 1,7-diaminoheptane (Sigma-Aldrich).

#### 2.6.3. Derivatization of Extracts and Standards

Derivatization of BAs was conducted according to the method described by Eerola, et al. [39]. One milliliter of extract or standard solution prepared as aforementioned was mixed with 200 μL of 2 M sodium hydroxide and 300 μL of saturated sodium bicarbonate (all from Sigma-Aldrich). Two milliliters of dansyl chloride (Sigma-Aldrich) solution (10 mg/mL) in acetone were added to the mixture and incubated at 40 °C for 45 min. The residual dansyl chloride was removed by adding 100 μL of 25% ammonium hydroxide and incubating for 30 min at 25 °C. Using acetonitrile, the mixture was adjusted to a final volume of 5 mL and centrifuged at 3000× *g* for 5 min. After filtration using 0.2 μm pore-size filters (Millipore), the filtered supernatant was kept at 4 °C until further analysis using HPLC.

#### 2.6.4. Chromatographic Separations

Chromatographic separation of BAs was conducted according to the method previously developed by Eerola, et al. [39] and modified by Ben-Gigirey, et al. [40]. An HPLC unit (YL9100, YL Instruments Co., Ltd., Anyang, Korea) equipped with a UV/vis detector (YL Instruments) and Autochro-3000 data system (YL Instruments) was used. For chromatographic separation, a Nova-Pak C_18_ 4 μm column (150 mm × 4.6 mm, Waters, Milford, MA, USA) held at 40 °C was utilized. The mobile phases were 0.1 M ammonium acetate dissolved in deionized water (solvent A; Sigma-Aldrich) and acetonitrile (solvent B; SK chemicals, Ulsan, Korea) adjusted to a flow rate of 1 mL/min with a linear gradient starting from 50% of solvent B reaching 90% by 19 min. A 10 μL sample was injected and monitored at 254 nm. The limits of detection were approximately 0.1 μg/mL for all BAs in standard solutions and bacterial cultures, and about 0.1 mg/kg for all BAs in food matrices [42].

### 2.7. Gene Expression Analyses in Bacterial Cultures and Cheonggukjang

#### 2.7.1. RNA Extraction and Reverse Transcription

Expression analysis of tyrosine decarboxylase gene (*tdc*) involved RNA extraction from bacterial cultures (for in vitro experiments) and *Cheonggukjang* samples (viz., *Cheonggukjang* groups prepared through fermentation; for in situ fermentation experiments) with a Ribo-Ex Total RNA isolation solution (Geneall, Seoul, Korea). The extraction was conducted according to the manufacturer’s instructions with minor modifications as follows. To prepare bacterial culture for in vitro gene expression analysis, a loopful (10 µL) of glycerol stock of each enterococcal strain was inoculated in 5 mL of MRS broth supplemented with 0.5% L-histidine monohydrochloride monohydrate, L-tyrosine disodium salt hydrate, L-ornithine monohydrochloride, L-lysine monohydrochloride (pH 5.8), and 0.0005% pyridoxal-HCl (all from Sigma-Aldrich) and incubated at 37 °C for 48 h. Approximately 100 μL of the broth culture was then transferred to another tube containing 5 mL of the same medium and incubated under the same conditions. As for *Cheonggukjang* samples, 10 g of *Cheonggukjang* were gently mixed with 40 mL of phosphate buffer in a sterile bag, and the liquid part was collected. Subsequently, 3 mL of the bacterial culture or all liquid part of the mixture were immediately transferred into a 50 mL conical tube and centrifuged at 10,000× *g* at 4 °C for 5 min. After removing the supernatant, the pellet was suspended with 7 mL of phosphate buffer and centrifuged under the same conditions. Then, the pellet was homogenized with 800 μL of Ribo-Ex reagent in a bacterial lysing tube (Lysing Matrix B; MP Biomedicals, Santa Ana, CA, USA) using a Precellys 24 homogenizer (Bertin Technologies, Montigny, France) with two cycles for 30 s at 6800 rpm, pausing for 90 s between cycles. Approximately 200 μL of chloroform were added to the lysate, vortexed, and centrifuged at 10,000× *g* for 1 min. Approximately 400 μL of the supernatant were mixed with 600 μL of chilled absolute ethanol. The mixture was reacted at −70 °C for 15 min and purified with a Nucleospin RNA kit (Macherey-Nagel, Düren, Germany) according to the manufacturer’s instructions. The quality of the extracted RNA was evaluated using a NanoDrop 1000 spectrophotometer (Thermo Fisher, Waltham, MA, USA).

ReverTra Ace qPCR RT Master Mix with gDNA Remover kit (Toyobo, Osaka, Japan) containing reverse transcriptase, RNase inhibitor, oligo (dT) primers, random primers, and deoxynucleoside triphosphates (dNTPs) was used to synthesize cDNA from 1 μL of extracted RNA according to the manufacturer’s instructions. Reverse transcription was conducted under the following conditions: 37 °C for 15 min, 50 °C for 5 min, and 98 °C for 5 min. After the reaction, the resulting cDNA was stored at −70 °C until quantitative PCR analysis.

#### 2.7.2. Quantitative PCR Analysis

As designed by Kang, et al. [43], *q-tdc* F (5′-AGACCAAGTAATTCCAGTGCC-3′) and *q-tdc* R (5′-CACCGACTACACCTAAGATTGG-3′) primers were used for the quantitation of *tdc* gene expression by *E. faecium*. The primers for reference genes including *q-gap* F (5′-ATACGACACAACTCAAGGACG-3′) and *q-gap* R (5′-GATATCTACGCCTAGTTCGCC-3′) [34], along with *tufA*-RT F (5′-TACACGCCACTACGCTCAC-3′) and *tufA*-RT R (5′-AGCTCCGTCCATTTGAGCAG-3′) [44] were used for the normalization of *tdc* gene expression. The efficiency of each set of primers for reverse transcription quantitative polymerase chain reaction (RT-qPCR) was determined by the following equation: *E* = 10 ^(−1/*S*)^ − 1, where *E* is the amplification efficiency and *S* is the slope of standard curves generated through threshold cycle (Ct) values of serial dilutions of cDNA obtained from reverse-transcription of RNA from *E. faecium* KCCM 12118.

For the RT-qPCR analysis of *tdc* gene expression in bacterial cultures and *Cheonggukjang* samples, 5 μL of a 10-fold diluted cDNA were added to 15 μL of a master mix containing 10 μL of Power SYBR Green PCR Master Mix (Applied Biosystems, Foster City, CA, USA), 3 μL of RNase free water, and 1 μL of each primer (forward and reverse; 500 nM). Subsequently, thermal cycling was conducted using an Applied Biosystems 7500 Real-Time PCR system (Applied Biosystems) with the thermal cycling conditions programmed as follows: initial denaturation at 95 °C for 10 min; 40 cycles at 95 °C for 15 s (denaturation step), and 60 °C for 60 s (annealing and elongation steps, unless otherwise mentioned). Annealing and elongation conditions for primer *tufA*-RT were set at 55 °C for 60 s. Melting curve analysis was conducted using the RT-PCR system to confirm the specificity and to analyze the amplified products. Ct values were detected when the emissions from fluorescence exceeded the fixed threshold automatically determined by thermocycler software. Relative expression of *tdc* genes was further calculated by the 2^−(ΔΔct)^ method, normalized to the expression levels detected in *E. faecium* KCCM 12118 (refer to Figure 2) or *Cheonggukjang* groups fermented at 37 °C (refer to Figure 6), and expressed as n-fold differences to compare gene expression levels in different bacterial cultures and *Cheonggukjang* samples.

### 2.8. Statistical Analyses

Data were presented as means and standard deviations of duplicates or triplicates. All measurements on retail products were performed in triplicates, while the other experiments were conducted in duplicate. The significance of differences was determined by one-way analysis of variance (ANOVA) with Fisher’s pairwise comparison module of the Minitab statistical software, version 17 (Minitab Inc., State College, PA, USA), and differences with probability (*p*) value of <0.05 were considered statistically significant.

## 3. Results and Discussion

### 3.1. Physicochemical Properties of Retail Cheonggukjang Products

Physicochemical and microbial properties as well as BA content in retail *Cheonggukjang* products were analyzed to estimate the contributing factors to BA content (particularly tyramine) in *Cheonggukjang* (Section 3.1, Section 3.2 and Section 3.3). Table 1 displays the physicochemical properties of *Cheonggukjang* products purchased from retail markets in the Republic of Korea. The pH ranged from 6.39 to 7.05, with an average pH of 6.84 ± 0.23 (mean ± standard deviation). The results were similar to the study conducted by Lee, et al. [45], which reported the average pH of *Cheonggukjang* to be 7.0 ± 0.8. Jeon, et al. [19] and Yoo, et al. [46] also reported the average pH of *Cheonggukjang* to be pH 6.07 ± 0.72 (range of pH 4.62–8.14) and pH 7.21 ± 0.59 (range of pH 5.89–7.95), respectively. Such differences in the pH of the *Cheonggukjang* products may be owing to different fermentation conditions [47] and/or fermentation metabolites [48]. The salinity of retail *Cheonggukjang* products ranged from 1.95 to 9.36% with an average salinity of 5.16 ± 2.78%. In comparison, Ko, et al. [18], Jeon, et al. [19], and Kang, et al. [49] reported the average salinity of *Cheonggukjang* to be 2.12 ± 1.66% (0.12–11.51%), 1.56 ± 1.19% (0.10–5.33%), and 3.51 ± 2.45 (1.64–8.39%), respectively. Though the salinity of the *Cheonggukjang* products was found to vary substantially, Ko, et al. [18] suggested that the large differences in *Cheonggukjang* salinities may be traced to the production process, as some methods utilize the addition of different amounts of salt to preserve the fermented soybean product. The water activity of retail *Cheonggukjang* products ranged from 0.919 to 0.973 with an average of 0.951 ± 0.019. In a previous study by Kim, et al. [47], the average water activity was found to be 0.962 ± 0.028 (0.857–0.991). Overall, the physicochemical properties of retail *Cheonggukjang* products analyzed in the current study were mostly similar to the values reported in previous studies. Although the results of the current study did not show any correlation between physicochemical properties and BA content (especially tyramine) based on linear regression analyses (data not shown), it is noteworthy that the ranges of the physicochemical parameters were within the specific conditions for the growth of *E. faecium*, which are as follows: pH, from 4 to 10 [50]; salinity, up to 7% [50]; water activity, above 0.940 [51].

### 3.2. Microbial Properties of Retail Cheonggukjang Products

Table 2 shows the microbial properties of retail *Cheonggukjang* products. The number of total mesophilic viable bacteria ranged from 8.54 to 9.81 Log CFU/g, with an average of 9.27 ± 0.45 Log CFU/g. Comparatively, Ko, et al. [18] and Jeon, et al. [19] reported the total counts of viable mesophilic bacteria of *Cheonggukjang* products to be 7.50 ± 1.01 Log CFU/g (5.30–9.98 Log CFU/g) and 9.65 ± 0.77 Log CFU/g (8.23–11.66 Log CFU/g), respectively. The wide range of total mesophilic viable bacteria may result from an insufficient standardization of *Cheonggukjang* manufacturing processes such as different fermentation materials and conditions [18,47]. *Enterococcus* spp. were detected at concentrations of 6.64–7.99 Log CFU/g, with an average of 7.17 ± 0.49 Log CFU/g. The number of lactic acid bacteria was found to be approximately 6.66–8.12 Log CFU/g, with an average of 7.09 ± 0.58 Log CFU/g (Table 2). For comparison, a study by Kang and Park [25] showed that *Enterococcus* spp. were detected in all 31 *Cheonggukjang* products at concentrations of 3.51–8.46 Log CFU/g, with an average of 5.95 ± 1.60 Log CFU/g. In the report, approximately 58% and 16.8% of the isolated *Enterococcus* strains were identified as *E. faecium* and *E. faecalis*, respectively. The presence of *E. faecium* in *Cheonggukjang* was also reported by Kang, et al. [26]. The reported results on the presence of *Enterococcus* spp. at high concentrations in *Cheonggukjang* concurred with the findings of the current study. As *E. faecium* has been reported as a pathogenic and/or tyramine-producing bacterium detected in some foods including Chinese and Japanese fermented soybean products, previous studies have mentioned that preventative measures are necessary to avoid contamination during the manufacturing of fermented foods [52,53,54,55]. The traditional *Cheonggukjang* production process may also be susceptible to contamination by harmful microbes owing to the reliance on rice straw containing *B. subtilis* for fermentation [56]. In fact, according to Heu, et al. [57], rice straw contains a variety of bacteria, including mesophiles, thermophiles, coliforms, and actinomycetes, as well as fungi. Moreover, as sterilization processes are not utilized in the manufacturing of *Cheonggukjang*, occasional contamination by tyramine-producing bacteria such as *E. faecium* may be present in the final product. The results of the current and previous studies suggest that further research appears to be necessary for the development of methods to inhibit *E. faecium* growth during the manufacturing of *Cheonggukjang* as well as other fermented soybean products described above.

### 3.3. BA Content of Retail Cheonggukjang Products

*Cheonggukjang* contains abundant BA precursor amino acids such as lysine, histidine, tyrosine, and phenylalanine [58]. The high amino acid content may pose a risk for conversion into BAs during *Cheonggukjang* fermentation. In the present study, tryptamine, *β*-phenylethylamine, putrescine, cadaverine, histamine, tyramine, spermidine, and spermine contents in retail *Cheonggukjang* products were detected at concentrations of 70.63 ± 44.74 mg/kg, 36.22 ± 29.55 mg/kg, 10.80 ± 5.07 mg/kg, 18.57 ± 9.08 mg/kg, 8.37 ± 2.40 mg/kg, 457.42 ± 573.15 mg/kg, 121.92 ± 19.69 mg/kg, and 187.20 ± 110.27 mg/kg, respectively (Table 3). A previous study suggested toxicity limits of 30 mg/kg for *β*-phenylethylamine, 100 mg/kg for histamine, and 100–800 mg/kg for tyramine in foods [21]. Therefore, the evaluation of the BA content of the *Cheonggukjang* products was continued with regard to the aforementioned BA intake limits with the exception of tyramine (at 100 mg/kg). Evaluation of *β*-phenylethylamine content in two *Cheonggukjang* products revealed that concentrations exceeded the recommended limit of 30 mg/kg, with one product exceeding the limit by a factor of approximately 3. Though the histamine content of all *Cheonggukjang* products was found to be below the recommended limit of 100 mg/kg, the tyramine content of three products exceeded the 100 mg/kg limit by factors of 2, 9, and 14, respectively. Other studies have reported similarly high concentrations of tyramine in *Cheonggukjang*. Ko, et al. [18] and Seo, et al. [20] reported the highest concentrations of tyramine in *Cheonggukjang* products, exceeding the recommended limit by factors of 19 and 9, respectively. Furthermore, Cho, et al. [59], Han, et al. [60], and Jeon, et al. [19] reported that *Cheonggukjang* products contained high concentrations of tyramine, which exceeded the recommended limit by up to factors of 5, 5, and 3, respectively. Altogether, as *β*-phenylethylamine and tyramine content of several *Cheonggukjang* products exceeded the recommended limits, overconsumption of such fermented soybean products may occasionally result in adverse effects on the body. Moreover, *Cheonggukjang* was found to contain other BAs enhancing the toxicity of *β*-phenylethylamine and tyramine. Therefore, further research remains necessary for precautionary measures and remedial methods to reduce the BA content of *Cheonggukjang* to ensure the safety of the fermented soybean food.

### 3.4. In Vitro BA Production by Enterococcus Strains Isolated from Retail Cheonggukjang Products

**Figure 1:**
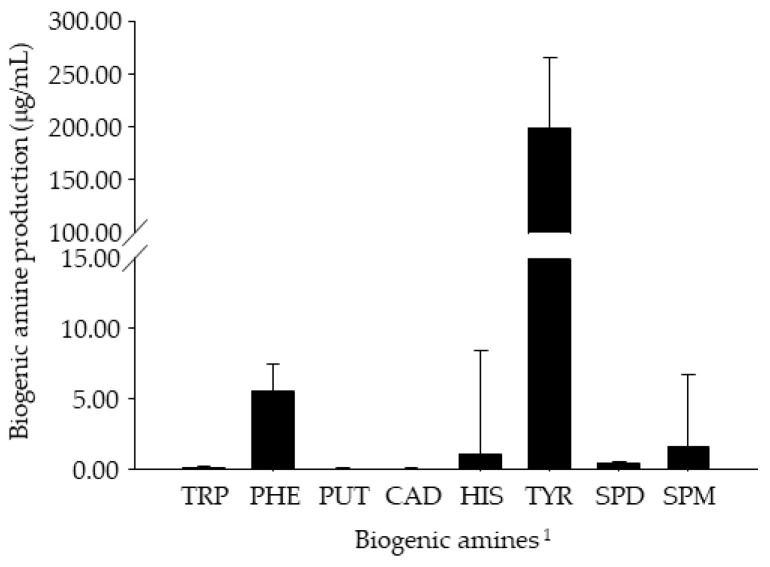
Biogenic amine (BA) production by *Enterococcus* strains (n = 169) isolated from retail *Cheonggukjang* products. Error bars indicate standard deviations calculated from duplicate experiments. ^1^ TRP: tryptamine, PHE: *β*-phenylethylamine, PUT: putrescine, CAD: cadaverine, HIS: histamine, TYR: tyramine, SPD: spermidine, SPM: spermine.

**Figure 2:**
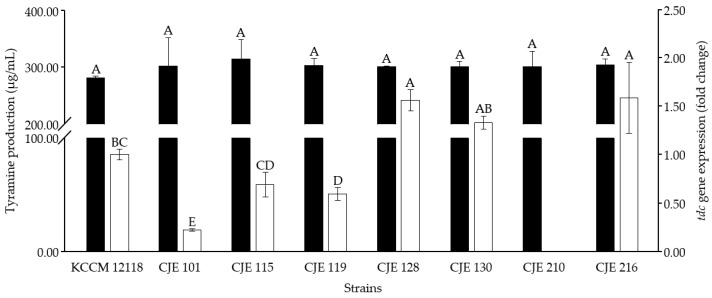
Comparison of tyramine production and *tdc* expression by *E. faecium* strains. ■: tyramine production, □: *tdc* gene expression. The expression levels observed in *E. faecium* strains isolated from retail *Cheonggukjang* products were normalized to that detected in *E. faecium* KCCM 12118 (type strain). The *tdc* gene expression was not detected in *E. faecium* strain CJE 210. Mean values followed by different letters are significantly different (*p* < 0.05). Error bars indicate standard deviations calculated from duplicate experiments.

Microbial decarboxylation of free amino acids is one of the main causing factors in the production of BAs, and various microorganisms, including *Bacillus*, *Clostridium*, Enterobacteriaceae, enterococci, *Lactobacillus*, and *Pseudomonas*, are capable of producing the decarboxylases responsible for the conversion of amino acids into BAs [21,61,62]. Considering previous studies in which *Enterococcus* spp. have been suggested to be responsible for tyramine accumulation in Chinese and Japanese fermented soybean products [52,53], the current study analyzed the BA production capabilities of 169 enterococcal strains isolated from retail *Cheonggukjang* products using an MRS broth-based assay medium. As shown in Figure 1, the production of tryptamine, *β*-phenylethylamine, putrescine, cadaverine, spermidine, and spermine was observed at concentrations lower than 10 µg/mL. Histamine production by 168 of the 169 strains was detected at quantities lower than 2 µg/mL; however, only one strain was capable of producing histamine at 96.06 µg/mL. Though tyramine production ranged from ND (not detected) to 315.29 µg/mL, 157 strains (about 93%) produced over 100 µg/mL. Through 16S rRNA sequencing, the seven strains (CJE 101, CJE 115, CJE 119, CJE 128, CJE 130, CJE 210, and CJE 216) that produced the highest levels of tyramine (301.14–315.29 μg/mL; refer to Figure 2) among the enterococcal strains were all identified as *E. faecium*. Novella-Rodríguez, et al. [63] suggested that the presence of Enterobacteriaceae or enterococci may result in the production of BAs in contaminated food products. Marcobal, et al. [64] demonstrated that *E. faecium* possesses a gene that codes an enzyme capable of L-tyrosine decarboxylation. According to Ibe, et al. [22], high levels of tyramine in *Miso* (a Japanese fermented soybean paste) products may partially result from tyramine production by *E. faecium*. Jeon, et al. [19] reported that *Enterococcus* spp. exhibited strong production of tyramine ranging from 0.41 μg/mL to 351.59 μg/mL in assay media. The author also found tyramine-producing *Bacillus* spp. (up to 123.08 μg/mL) and suggested that the species is one of the major tyramine producers in *Cheonggukjang* along with *Enterococcus* species based on the in situ fermentation experiment. Consequently, the present results suggest that *Enterococcus* spp. (particularly *E. faecium*) may be largely responsible for high tyramine concentrations in *Cheonggukjang*.

### 3.5. Selection of Tyramine-Producing E. faecium Strain for Cheonggukjang Fermentation Based on Tyrosine Decarboxylase Gene Expression In Vitro

In the current study, the efficiency of primer sets *q-tdc* (for the quantitation of *tdc* gene expression) along with *q-gap* and *tufA*-RT (for the normalization of *tdc* gene expression) was calculated to be 100.71%, 94.84%, and 95.03%, respectively. An amplification efficiency between 90 and 110% indicates that the results of gene expression obtained using RT-qPCR are reproducible [65].

The aforementioned primer sets were used to detect *tdc* gene expression by the seven *E. faecium* strains (CJE 101, CJE 115, CJE 119, CJE 128, CJE 130, CJE 210, and CJE 216) with the highest tyramine production in vitro as described in the previous section (note that the primer sets were also used for in situ gene expression analysis). Among the strains, *E. faecium* strain CJE 216 showed the highest expression level of *tdc* gene (Figure 2). Considering the highest *tdc* gene expression as well as tyramine production in vitro, the CJE 216 strain was selected as an inoculant for fermentation experiments in the next section.

### 3.6. Tyramine Production by E. faecium during Cheonggukjang Fermentation at Various Temperatures

#### 3.6.1. Changes in Physicochemical and Microbial Properties during *Cheonggukjang* Fermentation at Various Temperatures

**Figure 3:**
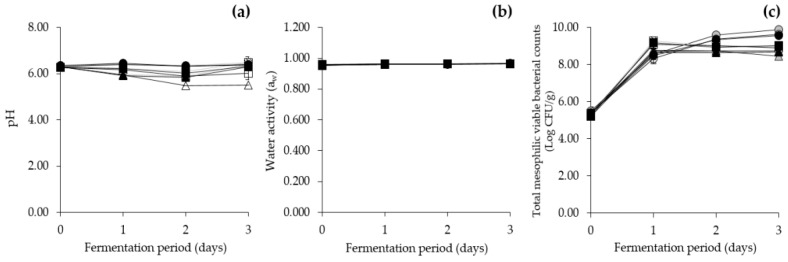
Physicochemical and microbial properties during *Cheonggukjang* fermentation at various temperatures. (**a**) pH, (**b**) water activity, (**c**) total mesophilic viable bacterial counts. ●: 25 °C, ■: 37 °C, ▲: 45 °C (inoculated with only *B. subtilis* KCTC 3135); ●: 25 °C, ■: 37 °C, ▲: 45 °C (inoculated with *B. subtilis* KCTC 3135 and *E. faecium* KCCM 12118); ○: 25 °C, □: 37 °C, Δ: 45 °C (inoculated with *B. subtilis* KCTC 3135 and *E. faecium* CJE 216). Error bars indicate standard deviations calculated from duplicate experiments.

As the results of the previous sections indicated that *E. faecium* was most likely one of the major contributing factors to high levels of tyramine in *Cheonggukjang*, in situ fermentation experiments were performed to empirically investigate the influence of *E. faecium* on tyramine content in *Cheonggukjang*. For the in situ fermentation experiments, three experimental groups of *Cheonggukjang* were used: control group inoculated with only *B. subtilis* KCTC 3135, and other two groups co-inoculated with the *B. subtilis* strain and each *E. faecium* strain (*E. faecium* KCCM 12118 or *E. faecium* CJE 216). Each group was further divided into three groups based on fermentation temperatures of 25 °C, 37 °C, and 45 °C (low-, intermediate-, and high-temperature groups, respectively). As shown in Figure 3, the changes in the physicochemical and microbial properties of *Cheonggukjang* were measured at 24-hour intervals for 3 days of fermentation. The pH of all *Cheonggukjang* groups was lowest on day 2, with progressively lower pH depending on the fermentation temperature, independent of which inoculum was used. The pH on day 2 of *Cheonggukjang* fermentation at 25 °C, 37 °C, and 45 °C ranged from pH 6.34 to 6.36, pH 5.90 to 6.09, and pH 5.49 to 5.88, respectively (Figure 3a). Loizzo, et al. [66] suggested that decarboxylases are produced by bacteria owing to a mechanism to neutralize acidic environments that restrict the growth of the bacteria. A previous study reported that low pH between 4.0 and 5.5 may result in the production of BAs [67]. Therefore, in this study, as the *Cheonggukjang* groups fermented at 45 °C (high-temperature group) resulted in a lower pH than other groups fermented at 37 °C and 25 °C (intermediate- and low-temperature groups, respectively), regardless of inoculum, the BA content was expected to be detected at the highest concentration among all *Cheonggukjang* groups. As for water activity, all *Cheonggukjang* groups remained within 0.95–0.97 during fermentation (Figure 3b).

The counts of total mesophilic viable bacteria, most probably attributed to *B. subtilis* inoculated, showed that microbial concentrations started from approximately 6 Log CFU/g on day 0 and remained at approximately 8–9 Log CFU/g throughout *Cheonggukjang* fermentation at all three temperatures, regardless of the presence or absence of *E. faecium* inoculum (Figure 3c). The total mesophilic viable bacteria in *Cheonggukjang* increased as fermentation temperature decreased; however, on day 1, those in the groups fermented at 25 °C and 45 °C showed growth up to 8 Log CFU/g, while those in the groups fermented at 37 °C exhibited the highest counts at 9 Log CFU/g. The results concurred with a previous finding that 37 °C is the optimal in situ growth temperature for *B. subtilis* during *Cheonggukjang* fermentation [29]. Similarly, Mann, et al. [68] reported the optimal in vitro growth temperature for *B. subtilis* strains isolated from *Cheonggukjang* to be 37 °C.

**Figure 4:**
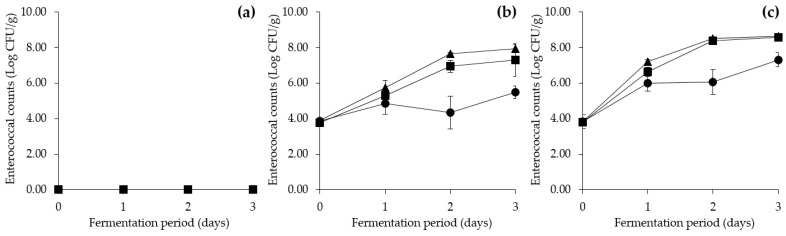
Effect of fermentation temperature on enterococcal counts in *Cheonggukjang* inoculated with (**a**) *B. subtilis* KCTC 3135, (**b**) *B. subtilis* KCTC 3135 and *E. faecium* KCCM 12118, and (**c**) *B. subtilis* KCTC 3135 and *E. faecium* CJE 216. ●: 25 °C, ■: 37 °C, ▲: 45 °C. Error bars indicate standard deviations calculated from duplicate experiments.

The enterococcal count in *Cheonggukjang* co-inoculated with *E. faecium* KCCM 12118 at approximately 4 Log CFU/g (and *B. subtilis* KCTC 3135 at 6 Log CFU/g as well) increased by 1.63 Log CFU/g, 3.52 Log CFU/g, and 4.06 Log CFU/g after 3 days of fermentation at 25 °C, 37 °C, and 45 °C, respectively (Figure 4). In *Cheonggukjang* co-inoculated with *E. faecium* CJE 216 at 4 Log CFU/g (and *B. subtilis* KCTC 3135), enterococcal count increased at all fermentation temperatures of 25 °C, 37 °C, and 45 °C by 3.48 Log CFU/g, 4.78 Log CFU/g, and 4.80 Log CFU/g, respectively, by day 3. *Enterococcus* spp. were not detected in the control group for the duration of the fermentation period. The results displayed progressively higher enterococcal counts that increased alongside rising fermentation temperatures with the highest enterococcal counts in *Cheonggukjang* fermented at 45 °C (high-temperature group). The findings were comparable to a previous study by Morandi, et al. [69], which showed that lower fermentation temperatures weakened *E. faecium* growth as the reported generation time at 25 °C was nearly two times longer than at 37 °C. *E. faecium* has been reported to display active growth within the temperature range of 37–53 °C, with an optimal growth temperature of 42.7 °C [50,70]. The current and previous studies, therefore, indicate that the use of high fermentation temperatures such as 45 °C may enhance *E. faecium* growth, thereby increasing the potential for high tyramine production during *Cheonggukjang* fermentation.

#### 3.6.2. Effect of Fermentation Temperature on Tyramine Content in *Cheonggukjang*

**Figure 5:**
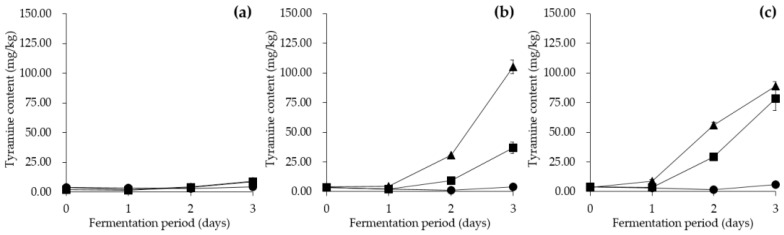
Effect of fermentation temperature on tyramine content in *Cheonggukjang* inoculated with (**a**) *B. subtilis* KCTC 3135, (**b**) *B. subtilis* KCTC 3135 and *E. faecium* KCCM 12118, and (**c**) *B. subtilis* KCTC 3135 and *E. faecium* CJE 216. ●: 25 °C, ■: 37 °C, ▲: 45 °C. Error bars indicate standard deviations calculated from duplicate experiments.

The tyramine content of *Cheonggukjang* co-inoculated with either *E. faecium* KCCM 12118 or *E. faecium* CJE 216, along with *B. subtilis* KCTC 3135, was measured during fermentation, as seen in Figure 5. The tyramine content of the control group without *E. faecium* was detected at concentrations that did not exceed 10 mg/kg in all fermentation conditions (Figure 5a). In contrast, other groups with *E. faecium* contained higher levels of tyramine, which indicated that *E. faecium* was capable of and responsible for producing tyramine in *Cheonggukjang*. In *Cheonggukjang* groups co-inoculated with *E. faecium* KCCM 12118, initial tyramine content increased by 0.78 mg/kg, 33.36 mg/kg, and 101.17 mg/kg at 3 days of fermentation at 25 °C, 37 °C, and 45 °C, respectively (Figure 5b). As for *Cheonggukjang* groups co-inoculated with *E. faecium* CJE 216, initial tyramine content increased by 1.59 mg/kg, 74.11 mg/kg, and 85.14 mg/kg at 25 °C, 37 °C, and 45 °C, respectively, by day 3 of fermentation (Figure 5c). All *Cheonggukjang* groups fermented at 25 °C contained the lowest tyramine concentrations at less than 10 mg/kg during the entire fermentation duration. However, at 45 °C, the *Cheonggukjang* group co-inoculated with *E. faecium* KCCM 12118 displayed an exceptionally high tyramine content detected at 105.13 ± 5.68 mg/kg, exceeding the recommended limit, as expected owing to the acidic pH described in Section 3.6.1. Both *E. faecium* strains appeared to continuously produce tyramine during *Cheonggukjang* fermentation at 37 °C and 45 °C (Figure 5b,c). The results of the current study are in agreement with findings reported by Kalhotka, et al. [71], which showed a stronger in vitro tyramine production by *E. faecium* incubated at 37 °C than at 25 °C. According to Morandi, et al. [69], *E. faecium* metabolic activity was detected to be higher at 37 °C than at 25 °C during milk fermentation. The previous studies have indicated that lower temperatures may reduce both metabolic activity and tyramine production of *E. faecium*. BA content during fermentation at higher temperatures may even reach dangerously high levels as reported by Kang, et al. [43]. In the same report, tyramine concentrations in *E. faecium*-inoculated *Cheonggukjang* fermented at 45 °C for 48 h increased (up to 698.67 mg/kg) during the fermentation period, and consequently exceeded the recommended limit for consumption. Jeon, et al. [19] also reported strong tyramine production by *Enterococcus* spp. during soybean fermentation at 37 °C. The report demonstrated that tyramine concentrations continued to increase as fermentation progressed. Given the results, safety precautions regarding the limitation of fermentation duration and temperature appear to be necessary as extended periods of fermentation as well as high fermentation temperatures may increase tyramine content in *Cheonggukjang* beyond the recommended safe limit for consumption. Besides, the results showing a lower tyramine content in *Cheonggukjang* during fermentation at lower temperatures coincide with the results in the previous section that displayed a reduction in enterococcal count alongside a decrease in fermentation temperature. Taken together, the present study indicates that lower fermentation temperatures inhibit enterococcal growth, thereby limiting acid production and maintaining low levels of tyramine in *Cheonggukjang*. Therefore, utilizing lower temperatures for *Cheonggukjang* fermentation may reduce the risks associated with *Enterococcus* growth and tyramine accumulation.

#### 3.6.3. Effect of Fermentation Temperature on *tdc* Gene Expression by *E. faecium* Strains in *Cheonggukjang*

**Figure 6:**
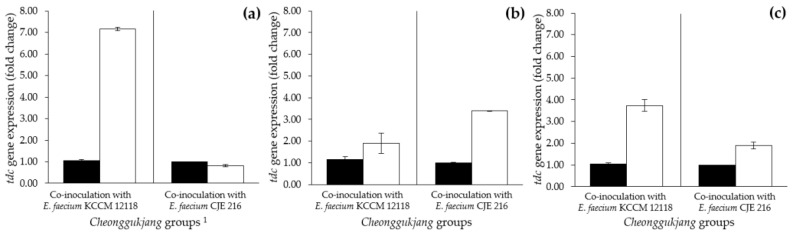
Effect of fermentation temperature on *tdc* expression by *E. faecium* strains in *Cheonggukjang* on (**a**) day 1, (**b**) day 2, and (**c**) day 3 of fermentation. ■: 37 °C, □: 45 °C. ^1^ *Cheonggukjang* groups were co-inoculated with *B. subtilis* (KCTC 3135) and *E. faecium* (KCCM 12118 or CJE 216) strains. The expression levels observed in groups fermented at 45 °C were normalized to those detected in the corresponding groups fermented at 37 °C. Expression of *tdc* gene was not detected in *Cheonggukjang* fermented at 25 °C. Error bars indicate standard deviations calculated from duplicate experiments.

The changes in *tdc* gene expression by tyramine-producing *E. faecium* strains were detected and quantified during fermentation of *Cheonggukjang* at 25 °C, 37 °C, and 45 °C. As *Cheonggukjang* is mostly fermented at 37 °C, the *tdc* gene expression detected in *Cheonggukjang* groups fermented at 45 °C was normalized to that detected in the corresponding groups fermented at 37 °C according to the *E. faecium* strains used as inoculants. In *Cheonggukjang* fermented at 25 °C, tyramine content continuously remained at concentrations lower than 10 mg/kg, and *tdc* gene expression by *E. faecium* KCCM 12118 and *E. faecium* CJE 216 was not detected in all *Cheonggukjang* groups. In contrast, the highest *tdc* gene expression by *E. faecium* KCCM 12118 was detected in *Cheonggukjang* fermented at 45 °C and was upregulated in the range of 1.90- to 7.15-fold throughout *Cheonggukjang* fermentation, compared with that in *Cheonggukjang* fermented at 37 °C (Figure 6a–c, left). As for *Cheonggukjang* fermented at 45 °C with *E. faecium* CJE 216, downregulation of *tdc* gene expression was observed at 0.82-fold on day 1, and the expression was then upregulated in the range of 1.90- to 3.39-fold thereafter (Figure 6a–c, right). Consequently, both tyramine content and *tdc* gene expression were highest in *Cheonggukjang* groups fermented at 45 °C (viz., high-temperature group). Nonetheless, the variation in detected *tdc* gene expression levels during fermentation did not necessarily reflect the tyramine content observed for *Cheonggukjang*. After one day of fermentation, the *Cheonggukjang* group with *E. faecium* CJE 216 fermented at 37 °C contained a lower tyramine content than at 45 °C; however, *tdc* gene expression was slightly higher at 37 °C as described right above. The results showed that there may be differences between gene expression level and enzyme activity (and products thereof). Glanemann, et al. [72] reported that, in vitro, the mRNA response levels do not necessarily reflect the protein response levels or enzyme activity. As previously suggested by Ladero, et al. [73], while the correlation between BA content and gene expression is not always linear, RT-qPCR remains a reliable method to detect and quantify BA-producing bacteria in food products. Similarly, in our preliminary tests conducted under different incubation conditions, tyramine production by *E. faecium* strains in an assay medium appeared to be insignificantly related to *tdc* gene expression level (data not shown). Therefore, utilizing HPLC analysis appears to be essential for the quantification of BA content and/or bacterial BA production in food samples [31,73,74]. When utilized in conjunction, the complementary methods, that is, HPLC and RT-qPCR, sufficiently allow for the quantitative analysis of both the BA content and tyramine-producing bacteria (including enterococci) in food products [24,31]. In the present study, the results derived from both techniques indicated that the fermentation of *Cheonggukjang* at high temperatures results in increased *tdc* gene expression and tyramine production. Therefore, low-temperature fermentation appears to be necessary to minimize both *tdc* gene expression and tyramine production by *Enterococcus* spp. and thereby ensure the safety of fermented soybean products.

## 4. Conclusions

The current study assessed the safety risk of tyramine in *Cheonggukjang*, diagnosed the microbial causative agent (i.e., *E. faecium*) responsible for high tyramine levels, and evaluated the impact of fermentation temperature on enterococcal growth (as well as acid production and *tdc* gene expression) and tyramine production. Of the retail *Cheonggukjang* examined, half of the products contained tyramine content that exceeded the recommended limit for safe consumption by up to a factor of approximately 14. *E. faecium* strains isolated from the retail *Cheonggukjang* products were highly capable of producing tyramine in assay media, which indicated that the species is principally, or at least partly, responsible for tyramine accumulation in the food.

During in situ fermentation at different temperatures, the tyramine content of *Cheonggukjang* groups co-inoculated with *B. subtilis* (used as an inoculant to ferment soybeans) and *E. faecium* (either isolated in this study or designated previously as the type strain) strains was highest at 45 °C, followed by 37 °C and 25 °C. On the other hand, the control group inoculated with only *B. subtilis* strain (without any *E. faecium* inoculants) had the lowest tyramine content at all fermentation temperatures, which supported the notion that *E. faecium* may be a key producer of tyramine in *Cheonggukjang*. Another implication of the results was that a lower fermentation temperature leads to a lower tyramine content below the recommended limit in *Cheonggukjang*, even though the tyramine content continually increases during fermentation. Therefore, low temperatures and a short fermentation duration may reduce the accumulation of tyramine caused by *E. faecium* growth in *Cheonggukjang*, thereby reducing the safety risks associated with consuming food with high BA concentrations.

## Figures and Tables

**Table 1:** Physicochemical properties of retail *Cheonggukjang* products.

Products ^1^	pH	Salinity (%)	Water Activity
CJ1	6.91 ± 0.03 ^2^	5.54 ± 0.09	0.948 ± 0.002
CJ2	6.84 ± 0.02	7.25 ± 0.06	0.919 ± 0.001
CJ3	6.87 ± 0.02	3.16 ± 0.06	0.968 ± 0.002
CJ4	6.99 ± 0.03	1.95 ± 0.09	0.973 ± 0.002
CJ5	7.05 ± 0.05	9.36 ± 0.59	0.944 ± 0.003
CJ6	6.39 ± 0.03	3.71 ± 0.34	0.954 ± 0.003
Average	6.84 ± 0.23	5.16 ± 2.78	0.951 ± 0.019

^1^ CJ: *Cheonggukjang*; ^2^ Mean ± standard deviation were calculated from triplicate experiments.

**Table 2:** Microbial properties of retail *Cheonggukjang* products.

Products ^1^	Total Mesophilic Viable Bacteria (Log CFU/g)	*Enterococcus* spp. (Log CFU/g)	Lactic Acid Bacteria (Log CFU/g)
CJ1	9.45 ± 0.06 ^2^	7.32 ± 0.03	7.45 ± 0.10
CJ2	9.04 ± 0.09	6.78 ± 0.14	6.66 ± 0.10
CJ3	9.81 ± 0.25	6.64 ± 0.01	6.70 ± 0.15
CJ4	9.57 ± 0.15	7.27 ± 0.03	6.96 ± 0.09
CJ5	8.54 ± 0.48	7.00 ± 0.08	6.67 ± 0.18
CJ6	9.20 ± 0.44	7.99 ± 0.04	8.12 ± 0.01
Average	9.27 ± 0.45	7.17 ± 0.49	7.09 ± 0.58

^1^ CJ: *Cheonggukjang*; ^2^ Mean ± standard deviation were calculated from triplicate experiments; CFU: colony-forming units.

**Table 3:** Biogenic amine (BA) content of retail *Cheonggukjang* products.

Products ^1^	BA Content (mg/kg) ^2^							
	TRP	PHE	PUT	CAD	HIS	TYR	SPD	SPM
CJ1	115.06 ± 19.72 ^A,3^	31.66 ± 3.82 ^B^	8.54 ± 3.45 ^BC^	22.66 ± 0.82 ^C^	8.60 ± 0.88 ^B^	222.25 ± 15.1 ^C^	137.88 ± 1.76 ^A^	292.99 ± 27.86 ^A^
CJ2	86.02 ± 3.12 ^B^	16.06 ± 2.24 ^B^	12.18 ± 0.73 ^B^	29.95 ± 0.82 ^A^	12.69 ± 0.28 ^A^	57.14 ± 8.06 ^D^	99.99 ± 4.25 ^C^	206.32 ± 23.82 ^B^
CJ3	118.27 ± 5.97 ^A^	27.22 ± 2.00 ^B^	18.33 ± 4.93 ^A^	26.58 ± 1.65 ^B^	6.50 ± 1.00 ^C^	80.83 ± 3.91 ^D^	96.82 ± 4.09 ^C^	201.63 ± 5.77 ^B^
CJ4	54.87 ± 3.18 ^C^	22.20 ± 7.06 ^B^	11.36 ± 2.28 ^B^	8.28 ± 0.54 ^F^	5.86 ± 0.37 ^C^	898.41 ± 79.43 ^B^	125.61 ± 4.64 ^B^	91.09 ± 24.03 ^C^
CJ5	47.85 ± 4.04 ^C^	24.48 ± 1.97 ^B^	11.61 ± 4.21 ^B^	13.57 ± 0.21 ^D^	8.65 ± 0.51 ^B^	61.98 ± 6.30 ^D^	125.98 ± 7.60 ^B^	305.05 ± 17.35 ^A^
CJ6	1.70 ± 2.94 ^D^	95.58 ± 46.97 ^A^	2.81 ± 1.77 ^C^	10.19 ± 0.50 ^E^	7.84 ± 0.33 ^B^	1424.04 ± 62.43 ^A^	145.18 ± 7.64 ^A^	26.20 ± 8.51 ^D^
Average	70.63 ± 44.74	36.22 ± 29.55	10.80 ± 5.07	18.57 ± 9.08	8.37 ± 2.40	457.42 ± 573.15	121.92 ± 19.69	187.20 ± 110.27

^1^ CJ: *Cheonggukjang*; ^2^ TRP: tryptamine, PHE: *β*-phenylethylamine, PUT: putrescine, CAD: cadaverine, HIS: histamine, TYR: tyramine, SPD: spermidine, SPM: spermine; ^3^ Mean ± standard deviation were calculated from triplicate experiments. Mean values in the same column followed by different letters (A–F) are significantly different (*p* < 0.05).
